# Biochemical, biophysical, and functional characterisation of the E3 ubiquitin ligase APC/C regulator CDC20 from *Arabidopsis thaliana*

**DOI:** 10.3389/fphys.2022.938688

**Published:** 2022-07-25

**Authors:** Maria-Alexa Cosma, Natalie L. Curtis, Charlotte Pain, Verena Kriechbaumer, Victor M. Bolanos-Garcia

**Affiliations:** Department of Biological and Medical Sciences, Faculty of Health and Life Sciences, Oxford Brookes University, Headington, OX, United Kingdom

**Keywords:** Spindle Assembly Checkpoint (SAC), CDC20, *Arabidopsis thaliana*, cell division, APC/C regulation, mitosis, chromosome segregation

## Abstract

The Anaphase Promoting Complex (APC/C), a large cullin-RING E3-type ubiquitin ligase, constitutes the ultimate target of the Spindle Assembly Checkpoint (SAC), an intricate regulatory circuit that ensures the high fidelity of chromosome segregation in eukaryotic organisms by delaying the onset of anaphase until each chromosome is properly bi-oriented on the mitotic spindle. Cell-division cycle protein 20 homologue (CDC20) is a key regulator of APC/C function in mitosis. The formation of the APC/C^CDC20^ complex is required for the ubiquitination and degradation of select substrates, which is necessary to maintain the mitotic state. In contrast to the roles of CDC20 in animal species, little is known about CDC20 roles in the regulation of chromosome segregation in plants. Here we address this gap in knowledge and report the expression in insect cells; the biochemical and biophysical characterisation of *Arabidopsis thaliana* (AtCDC20) WD40 domain; and the nuclear and cytoplasmic distribution of full-length AtCDC20 when transiently expressed in tobacco plants. We also show that most AtCDC20 degrons share a high sequence similarity to other eukaryotes, arguing in favour of conserved degron functions in AtCDC20. However, important exceptions were noted such as the lack of a canonical MAD1 binding motif; a fully conserved RRY-box in all six AtCDC20 isoforms instead of a CRY-box motif, and low conservation of key residues known to be phosphorylated by BUB1 and PLK1 in other species to ensure a robust SAC response. Taken together, our studies provide insights into AtCDC20 structure and function and the evolution of SAC signalling in plants.

## Introduction

The Spindle Assembly Checkpoint (SAC) is an intricate mechanism of cell division control that monitors the accurate segregation of the genetic material during mitosis. The SAC is evolutionary conserved in higher organisms, ranging from invertebrate and vertebrate animals to fungi and plants. The abnormal expression and/or loss of function of central protein components of the SAC such as BUB1 (Budding Uninhibited by Benzimidazoles 1), BUBR1 (Budding Uninhibited by Uenzimidazoles Uelated 1), BUB3 (Budding Uninhibited by Uenzimidazoles 3), MAD1 (Mitotic Arrest Deficient 1), MAD2 (Mitotic Arrest Deficient 2), and CDC20 (Cell Division Cycle protein 20) results in gross chromosome segregation defects (Kops et al., 2020; Lara-Gonzalez et al., 2021a; Cheeseman and Maiato, 2021; Uchida et al., 2021). In association with MAD2, BUBR1 and BUB3, CDC20 functions as a key regulator of the E3 ubiquitin ligase Anaphase Promoting Complex/Cyclosome (APC/C), a large macromolecular assembly that controls mitotic exit. In animals and yeasts, CDC20 plays essential roles in mitosis and meiosis, acting as a dual regulator (e.g., depending on the number of CDC20 molecules bound to the APC/C, CDC20 functions as an activator or an inhibitor of the APC/C) (Hartwell et al., 1973; Kapanidou et al., 2017; Barford, 2020; Lara-Gonzalez et al., 2021b; Piano et al., 2021). In these organisms, loss of CDC20 function results in mitotic inhibition and embryonic death (Kitagawa et al., 2002; Li et al., 2007; Cooper and Strich, 2011). In *Arabidopsis*, mutation of *Cdc20* results in aberrant meiotic spindle formation (Niu et al., 2015). Despite the overall conservation of SAC signalling across the animal and plant kingdoms, important species specific differences have been identified. For example, in *Arabidopsis*, the protein BUB3.1 is localised to unattached kinetochores following SAC activation but also to the phragmoplast, a plant cell structure that is formed during late cytokinesis, suggesting BUB3.1 may have other functions beyond SAC (Caillaud et al., 2009). Also in *Arabidopsis*, MAD1 and MAD2 have been reported to play important roles in the regulation of flowering and endopolyploidisation via competitive binding to the protein MODIFIER OF snc1-1 (MOS1), a negative regulator of plant immunity (Bao et al., 2014). Moreover, yeast and animals harbour a single copy of the *Cdc20* gene, whereas plants generally have multiple copies (Capron et al., 2003; Lima et al., 2010). Indeed, a thorough evolutionary study of *Cdc20* gene expansion identified multiple *Cdc20* genes in the *Charophyceae, Klebsormidiophyceae, Zygnematophyceae*, and land plant species, indicating a tendency of *Cdc20* gene expansion during streptophyte evolution (Lin et al., 2022) and suggesting that *Cdc20* gene duplications within-species were common during land plant evolution (Lin et al., 2022).

**FIGURE 1:**
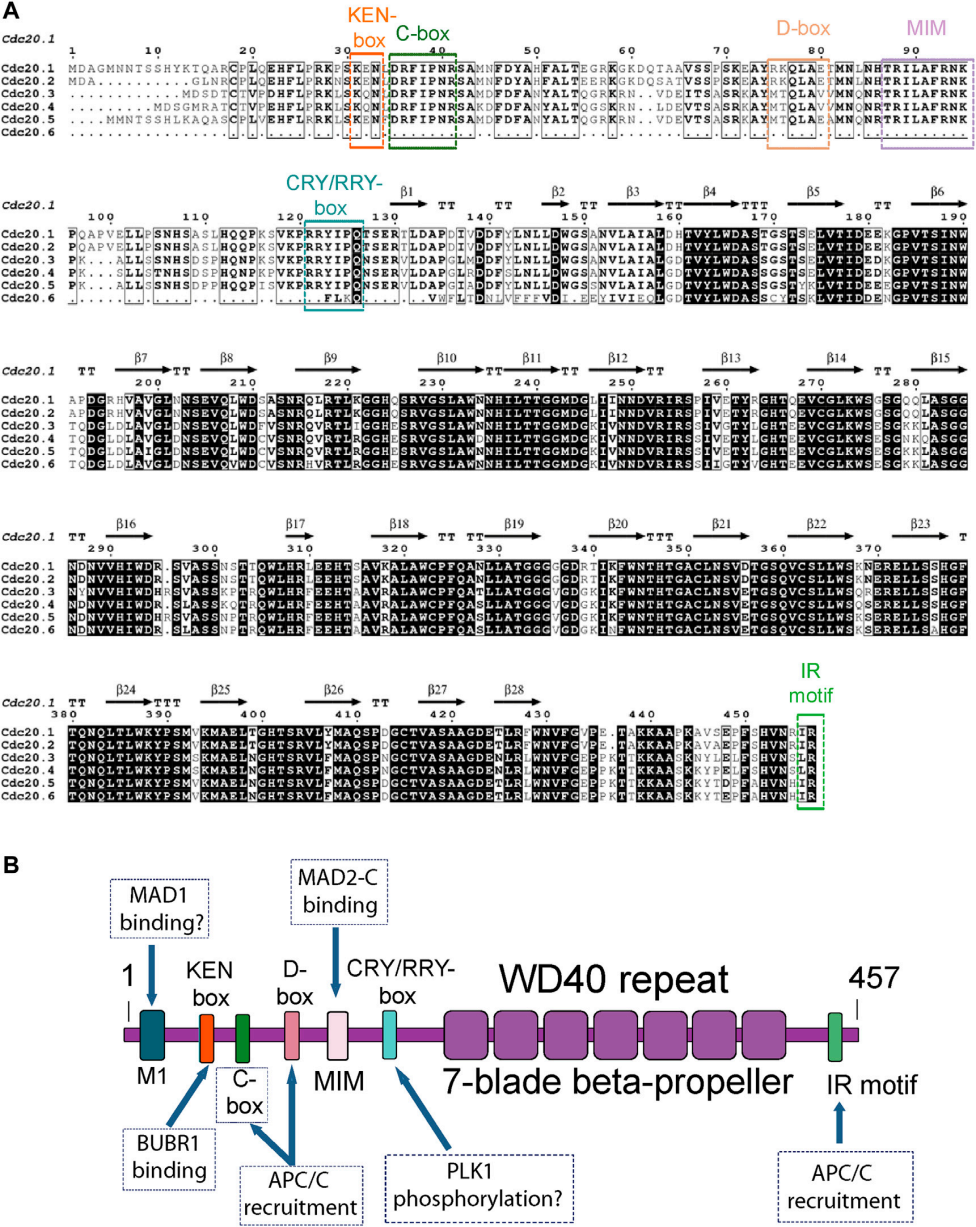
**(A)** Amino acid sequence alignment of the six AtCDC20 isoforms reported to date. The assignation of secondary structure elements was based on the 3D model structure of the AtCDC20.1 WD40 domain. The alignment was created with Clustal Omega (Sievers et al., 2011) and visualised with EsPript (Robert and Gouet, 2014). **(B)** Domain organisation and distribution of SLiMs of full length AtCDC20. The boxes indicate the known functions of the CDC20 SLiMs in the SAC. MIM refers to the MAD2-interacting motif. Plausible functions of other potential motifs of AtCDC20 are indicated with a question mark.

The *Arabidopsis thaliana* (*A. thaliana*) genome contains six *Cdc20* genes, *AtCDC20.1 (At4g33270), AtCDC20.2 (At4g33260), AtCDC20.3 (At5g27080), AtCDC20.4 (At5g26900), AtCDC20.5 (At5g27570),* and *AtCDC20.6 (At5G27945)* as shown in The Arabidopsis Information Resource (TAIR). The latter gene is predicted to encode for a truncated CDC20 protein that lacks the N-terminal region (Marrocco et al., 2009), encoding for a protein of 428 residues in length. The other five isoforms encode for larger proteins of slightly different sizes. *AtCDC20.1* and *AtCDC20.2* are located on chromosome 4 with the same orientation and separated from each other by a 1 kb intergenic region, whereas *AtCDC20.3, AtCDC20.4, AtCDC20.5,* and *AtCDC20.6* are clustered on chromosome 5, as shown in TAIR. *AtCDC20.1* and *AtCDC20.2* share a similar gene structure, consisting of five exons and four introns while *AtCDC20.3, AtCDC20.4,* and *AtCDC20.5* have no introns (Kevei et al., 2011). *AtCDC20.6* consists of three exons. The most remarkable differences between the AtCDC20 protein isoforms are the low conservation of amino acid residues of the N-terminal region and two small insertions present in two of the isoforms (Figure 1A). A multiple amino acid sequence alignment using the Clustal Omega program (Sievers et al., 2011; Figure 1A) indicated that AtCDC20.1 and AtCDC20.2 share 99% sequence identity, with 439 amino acid residues occupying identical positions, while AtCDC20.3, AtCDC20.4 and AtCDC20.5 share 85% identity amongst each other, with 384 residues occupying identical positions. Including the AtCDC20.6 isoform in the comparison with AtCDC20.3, AtCDC20.4, and AtCDC20.5 resulted in nearly 59% sequence identity amongst each other. In contrast, the proteins AtCDC20.1 and AtCDC20.2 share only 70% identity with that of AtCDC20.3, AtCDC20.4, and AtCDC20.5, with 322 residues occupying identical positions. Very recently, a functional variant of the human *Cdc20* gene that lacks the N-terminal segment and encodes only for the WD40 domain was identified by Tsang and Cheeseman (2021), rising the possibility the *AtCDC20.6 (At5G27945)* gene may also encode for a truncated but functional CDC20 protein.

The higher number of gene isoforms and divergence of functions of certain SAC proteins such as BUB3, MAD1, MAD2, and Monopolar Spindle 1 (MPS1) in plants compared to vertebrate and fungal lineages, prompted us to investigate the biochemical, biophysical, and functional features of CDC20 from *A. thaliana* (AtCDC20), a protein that is organised in a WD40 domain (also known as the WD or β-transducin repeat) and an N-terminal region that is largely of low structural complexity. In this contribution we report the overproduction of a C-terminal construct of the AtCDC20.1 isoform that encompasses the WD40 domain and Ile-Arg (IR) motif and extends until the C-terminal end of the native protein in a baculovirus expression system. For simplicity, we call this construct AtCDC20.1 WD40. We also report the biochemical and biophysical characterisation of this protein domain in aqueous solution; an analysis of AtCDC20.1 degradation signals (degrons) conservation and function; and the subcellular localisation of full length AtCDC20.1 transiently expressed in tobacco leaves. We show that full length AtCDC20.1 harbours several functional short linear motifs (SLiMs) (Figure 1B), most of which are highly conserved across animals and yeasts. Two notable exceptions are a sequence degenerated CRY-box degron and the lack of a putative MAD1 binding motif, as discussed below. We observed that AtCDC20.1 WD40 is a monomeric domain of thermal stability similar to that of other WD40 domains of similar size but different origin and function and that transient expression of full length AtCDC20.1 in tobacco leaves showed the existence of different subcellular pools, mostly in the nuclei and the cytoplasm. These findings support the view that important structural and functional features of CDC20, such as its recruitment to the Mitotic Checkpoint Complex (MCC) and the APC/C, are evolutionary conserved in animals, yeast and plants and that other features, such as CDC20 mode of physical interaction with MAD1 (if any) and the extent of CDC20 post-translational modifications by central SAC kinases, may vary significantly in plants. In summary, our study provides details of AtCDC20 WD40 overexpression; oligomerisation state and stability in aqueous solutions; the anticipated roles of conserved CDC20 degrons in *A. thaliana* cell division regulation and the evolution of SAC signalling in plants.

## Materials and methods

### AtCdc20.1 WD40 gene cloning and heterologous gene expression

The addition of a 5’ sequence encoding for a hexahistidine-tandem after the start codon was included to facilitate the metal binding purification of an AtCDC20.1 isoform fragment encompassing the WD40 domain (e.g., amino acid residues 120-457) using a baculovirus-based expression system. Following gene amplification by PCR, the *AtCdc20.1WD40* amplicon was purified and cloned into the BamHI and SalI restrictions sites of the pOET5 vector (Oxford Expression Technologies, OET) using standard molecular cloning protocols. The integrity of the *AtCdc20.1* gene construct in pOET5 was confirmed by DNA Sanger sequencing (Source Bioscience Sequencing, Cambridge) using in-house designed pOET5 sequencing primers. 1.5 × 10^6^ cells of *Sfodoptera frugiperda 9* (Sf9) cells were seeded in 2 ml of growth medium into 35 mm cell culture dishes and incubated at room temperature. Serum-free, antibiotic free medium (Gibco^®^ TC 100 Insect Medium, TC 100) of 1 ml was added in sterile tubes for each co-transfection. To each 1 ml of medium 5 μL of transfection reagent (baculoFECTIN, OET Ltd.), including a mock-transfection control, were added and mixed gently. Incubation occurred at room temperature for 30 min to allow the nanoparticle-DNA complex to form. After the incubation, the culture medium was removed and 1 ml of baculoFECTIN/DNA mixture added drop-wise into the centre of the dish of cells. The dishes were then incubated in a sandwich box overnight at 28°C. The next day, 1 ml of the insect cell culture growth medium supplied with 10% Fetal Bovine Serum (FBS; labtech.com) was added to each dish so that each dish had 2 ml of medium and the incubation was continued at 28°C for four more days. The 2 ml culture medium was then harvested and stored at 2–8°C.

The seed stock of recombinant viruses (P0) was used to amplify and produce a high titre P1 virus stock in serum-free ESF 921 media (Expression Systems, LLC). The virus titre was determined by plaque-assay as per instructed in the Oxford Expression Technologies User Guide 2016.

**FIGURE 2:**
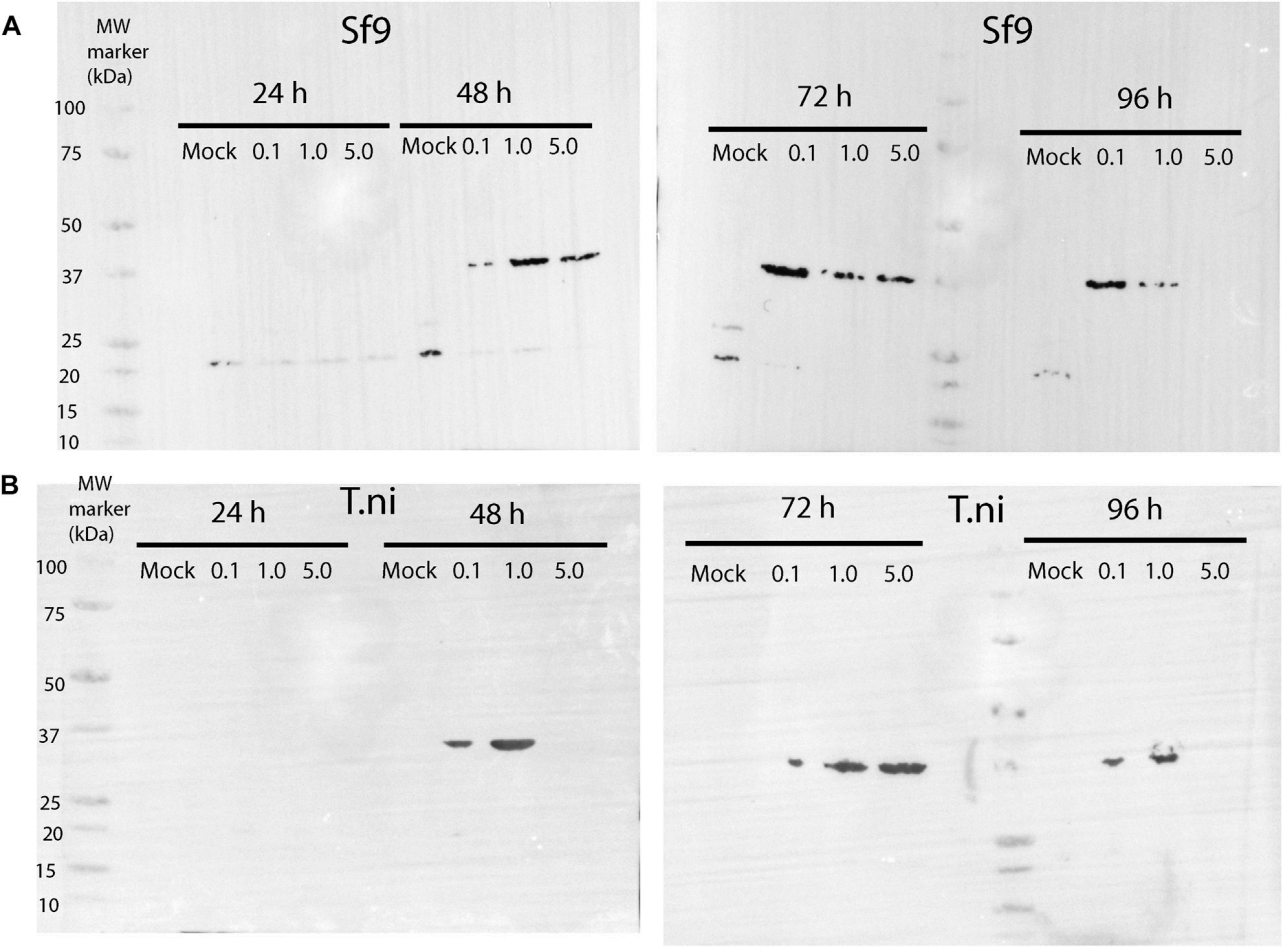
Western blot showing the time-course expression of AtCDC20.1 WD40 in Sf9 **(A)** and T.ni **(B)** cells. In each case, three different MOIs, 0.1, 1.0, and 5.0, were tested. Non-infected cells (e.g., mock) were used as a control.

For AtCDC20.1 WD40 small-scale protein production trials, the cell lines Sf9 and *Trichoplusia ni* (T.ni) at a cell density of 1.5 × 10^6^ cells/ml and 1.0 × 10^6^ cells/ml, respectively, were infected with the P1 virus stock at 0.1, 1.0, and 5.0 Multiplicity of Infection (MOI) titres. Samples of 1 ml from the different MOIs were collected after 24, 48, 72 and 96 h and harvested by centrifugation at 12,000 rpm for 10 min. The pellet and supernatant were stored at -20°C for further analyses. To breakdown the insect cells a lysis buffer solution consisting of 50 mM Tris pH 8.0, 5 mM β-mercaptoethanol, 100 mM KCl, 1% Nonidet P-40, cOmplete™, EDTA-free protease inhibitor cocktail tablets (Roche) was used. Cells membrane fragments and other debris were removed by centrifugation at 12,000 rpm, at 2–8°C for 15 min. An aliquot of the supernatant and the pellet was taken as the ‘soluble’ and ‘insoluble’ fraction, respectively for Sodium Dodecyl Sulphate Electrophoresis (SDS-PAGE) and Western Blotting analysis. For large-scale expression, 4 L of Sf9 insect cells in culture at 1.5 × 10^6^ cells/ml were infected and incubated at 28°C according to the optimised conditions found in the small-scale time-course expression tests (see Figure 2 for details). After 3 days incubation, the cells were harvested by centrifugation at 4,000 rpm, 2–8°C for 20 min (Beckman, J2-21) and the pellets washed with Phosphate Buffered Saline (PBS) buffer and stored at −20°C. Cell lysis was carried out as described above. Intact cells, cell membrane fragments, and other debris were removed by centrifugation at 12,000 rpm, 2–8°C for 45 min (Beckman, J2-21). Protein purification was carried out using the ÄKTA™ Start technology (GE Healthcare). Computational physicochemical parameters of the molecularly engineered protein such as pI and molecular mass were estimated using the ProtParam tool (Gasteiger et al., 2005; http://web.expasy.org/protparam).

### Western blotting

The primary antibody human CDC20 (AbCam 26483) was used to detect AtCDC20.1 WD40. We anticipated this antibody to work because the epitope is derived from within residues 450 to the C-terminal region and mapped onto a region of the WD40 domain that is highly conserved in both species (see Supplementary Figure S1A for details). An anti-rabbit alkaline phosphatase (AP)-IgG conjugate was used as the secondary antibody. Both primary and secondary antibodies were diluted according to supplier’s recommendations. Following three times of washes with TBST, colourimetric AP system (NBT/BCIP Substrate solution, Thermo Scientific Ltd.) was used for the chemiluminescence detection of AtCDC20.1 WD40 on the blot. AtCDC20.1 WD40 expression detection was also confirmed using an anti-histidine tag antibody conjugated to horse radish peroxidase (HRP) (AbCam, Cambridge). The colourimetric Clarity Western ECL Substrate (BioRad) was used for the chemiluminescence detection of histidine-tagged AtCDC20.1 WD40 on the blot (data not shown).

### AtCDC20.1 protein purification

Ni^2+^-IMAC was performed at 4°C in an ÄKTA Start system using the supernatant from the lysis and one HisTrap HP 5 ml column (GE Healthcare) previously equilibrated in binding buffer. The eluate was fractionated and collected in 5 ml fractions and analysed by SDS-PAGE. Pure fractions obtained from Ni^2+^-IMAC were pooled and used to perform preparative size exclusion chromatography (SEC) with a HiLoad 26/600 Superdex 200 pg column (GE Healthcare). The Superdex 200 pg column was pre-equilibrated with two column volumes of SEC buffer (e.g., 20 mM Tris, 200 mM NaCl, pH 8.0) prior to the loading of the sample. The flow-through was fractionated and peak fractions were analysed by SDS-PAGE using 4–12% BisTris precast gels (Invitrogen). The Precision Protein Plus Molecular Weight Marker (BioRad) was used as the protein molecular mass marker. Pure protein fractions were combined and stored for short storage at 4°C and for long term storage at −80°C.

### Biochemical and biophysical characterisation

Far-UV Circular Dichroism (CD) spectra were recorded on a Jasco J-815 Spectropolarimeter equipped with a computer-controlled Peltier temperature control unit. Spectra were recorded at 20°C in a quartz cuvette (Helma Analytics) of 0.1 cm path length, in the 260–190 nm range using an acquisition time of 1 s, step size of 0.5 nm, 1 nm bandwidth, scan speed at 100 nm/min and data averaged over four scans. The protein concentration of AtCDC20.1 WD40 that was used for the far-UV CD data collection was 0.5 mg/ml. The following buffers were used to define the stability of the domain at different pH: 40 mM sodium acetate; pH 4.0; 40 mM sodium acetate pH 5.0; 40 mM sodium phosphate pH 6.0; 40 mM sodium phosphate pH 7.0; 40 mM sodium phosphate pH 8.0; 40 mM sodium tetraborate pH 9.0.

Analytical Size Exclusion Chromatography coupled with Multi-Angle Light Scattering (SEC-MALS) was performed at 20°C using the Wyatt Heleos 8 light scattering (LS) detector and Wyatt Optilab rEX RI monitor linked to a Shimadzu HPLC system containing a LC-20AD pump, SIL-20A Autosampler and a SPD20A UV/Vis detector. A Superdex 75 HR10/30 column (GE Healthcare) was equilibrated in 25 mM Tris pH 8.0, 150 mM NaCl, 5 mM β-mercaptoethanol at a flow rate of 0.5 ml/min and 100 μL of 1 mg/ml AtCDC20.1 WD40 protein was injected to the column. The entire elution profile was used for the determination of the molecular mass using the Astra software (Wyatt Technology). SEC-MALS is used for the absolute definition of the molecular mass and oligomeric state of a protein in solution. The technique utilises a SEC column, which serves as the fractionation tool of the system, coupled with a LS, a Refractive Index (RI) and a UV detector. The LS detector monitors the excess of scattering light, the RI detector records the refractive index variances and the UV detector monitors the absorbance at 280 nm (Folta-Stogniew, 2006). The SEC-MALS technique is based on the principle that the weight-average molar mass and the molecular concentration of the molecule under investigation are directly proportional to the excess scattered light. The size of the molecule is determined by the proportional relation of scattering angle and is expressed in radius of gyration (Rg). Other size-related data that are generated from SEC-MALS analysis include the virial coefficient A2, which is a measurement of the solubility of the molecule in solution. The molar mass was calculated using either the forward monitor or laser monitor correction modes.

### Plant material and transient gene expression in tobacco leaves

For Agrobacterium-mediated transient expression, tobacco plants (*Nicotiana tabacum* SR1 cv Petit Havana) were grown in the glasshouse for 5 weeks with 14 h light and 10 h dark. Transient expression was carried out according to Sparkes et al. (2006). In brief, each construct was introduced into the *Agrobacterium* strain GV3101 by heat shock. Transformants were inoculated into 3 ml of YEB medium (5 g of beef extract, 1 g of yeast extract, 5 g of sucrose and 0.5 g of MgSO_4_^**.**^7H_2_O per L) with 50 μg/ml spectinomycin and 25 μg/ml rifampicin. The bacterial cultures were shaken overnight at 25°C, 1 ml of the bacterial culture was pelleted by centrifugation at 2,200 *g* for 5 min at room temperature. The pellet was washed twice with 1 ml of infiltration buffer (50 mM MES, 2 mM Na_3_PO_4_^**.**^12H_2_O, 0.1 mM acetosyringone and 5 mg/ml glucose) and then resuspended in 1 ml of infiltration buffer. The bacterial suspension was diluted to a final OD_600nm_ of 0.1 and carefully pressed through the stomata on the lower epidermal surface using a 1 ml syringe. Infiltrated plants were incubated for 72 h before imaging.

### Confocal microscopy

Images were taken using a Zeiss 880 laser scanning confocal microscope with 9,100/1.46 numerical aperture DIC M27 Elyra oil immersion objective. For imaging of the green/red fluorescent protein (GFP/RFP) combinations, samples were excited using 488 and 561 nm laser lines in multitrack mode with line switching. Signals were collected using the high-resolution Airyscan detector (Zeiss, Oberkochen, Germany) with emission wavelength of 523 nm for GFP and 579 nm for RFP. Images were edited using the ZEN image browser (Zeiss).

## Results

### Most AtCDC20 but not all SLiMs are conserved

We first investigated the extent of conservation of functional motifs in the six CDC20 isoforms from *A. thaliana* that have been annotated in TAIR and compared them to CDC20 found in animal and yeast orthologues. To this aim, multiple amino acid sequence alignments were carried out.

As shown in Figure 1A, the six AtCDC20 isoforms show a high amino acid sequence conservation of the C-terminal region encompassing the WD40 domain and IR motif. The high conservation of this motif extends to the C-terminal region of CDC20 from animals and yeast (Supplementary Figure S1A) and across plant species (Supplementary Figure S2).

The N-terminal segment of AtCDC20.1 is a region encompassing approximately 118 amino acid residues predicted to harbour short alpha-helical segments connected by a larger region of low structural complexity. Indeed, secondary prediction analyses using JPred (Drozdetskiy et al., 2015; Supplementary Figure S1B) predicted the presence of two short α-helices in the N-terminal region spanning residues 1-118. α-helix 1 is predicted to encompass residues 46-53 and α-helix 2, residues 71-81. These α-helices are flanked by large regions predicted to be disordered. Although the human CDC20 N-terminal region is predicted to contain two more short α-helices near the very N-terminal end (Supplementary Figure S1C) overall, the prediction was the same: a large N-terminal segment that is predominantly of low structural complexity. The lack of regular secondary structure of the N-terminal region of CDC20 is the reason this protein region was prone to spontaneous degradation when full length CDC20 was overexpressed in isolation (data no shown). The multiple sequence analysis revealed that key CDC20 SLiMs with important roles in the SAC are highly conserved in the *A. thaliana* CDC20 isoforms. Typically, these SLiMs mediate protein-protein interactions that control the timely degradation of CDC20 to drive SAC signalling (Davey and Morgan, 2016; Barford, 2020; Elowe and Bolanos-Garcia, 2022; Hartooni et al., 2022). In AtCDC20.1 these include the amino acid residues that define the KEN- box, K_31_E_32_N_33_; the C-box, D_35_R_36_F_37_I_38_P_39_N_40_R_41_; the destruction box (D-box), R_75_K_76_Q_77_L_78_A_79_E_80_T_81_,M_82_N_83_; the MAD2 interacting motif (MIM), R_88_I_89_L_90_A_91_F_92_P_96_; a degenerate CRY degron box, RRYIPQ, R_121_R_122_Y_123_I_124_P_125_Q_126_; and the residues that define the C-terminal IR motif, I_456_R_457_ (Figures 1A,B).

In contrast to the N-terminal region, which harbours several degrons, the C-terminal residues of all six AtCDC20 isoforms reported to date are largely organised in one domain containing the seven-blade β-propeller that defines the CDC20 WD40 fold followed by a very short segment -also predicted to be of low structure complexity- that contains one additional SLiM, the IR motif (Figure 1B). The β-propeller WD40 repeat and the terminal IR motif of AtCDC20.1 and AtCDC20.2 are key to CDC20 functions in the SAC as they are required for the interaction of CDC20 with the APC/C subunit APC10 and to BUB3.1 and BUBR1/MAD3 (Kevei et al., 2011). Interestingly, AtCDC20.3 (442 amino acid residues in length) and AtCDC20.4 (444 residues) contain a Leu-Arg (LR) sequence rather than IR. However, given the high physicochemical and structural similarity between Isoleucine and Leucine, it can be anticipated a high conservation of function in the C-terminal IR and LR motifs of AtCDC20 isoforms, including a role in APC/C binding. The C-box is another protein motif required for the binding of CDC20 to the APC/C core. A cross examination of the amino acid sequences of the six AtCDC20 isoforms revealed a high sequence conservation of the C-box motif, strongly suggesting than in *A. thaliana* this SLiM indeed contributes to the physical interaction of CDC20 with the APC/C complex to drive mitosis regulation.

At the same time, there are important differences in *A. thaliana* CDC20 compared to animals and yeast. In the latter organisms, the CDC20 region preceding the KEN-box motif contains a MAD1 binding motif that is rich in amino acid residues of basic nature. In human CDC20, this motif is defined by the residues R_27_W_28_Q_29_R_30_K_31_ (Ji et al., 2017; revised by; Luo et al., 2018). In contrast, all AtCDC20 isoforms lack a consensus MAD1 binding motif. However, two polar residues (R_26_K_27_ in AtCDC20.1) are conserved in five of the six AtCDC20 isoforms and these are preceded by other fully conserved residues (EHFLP) in AtCDC20 isoforms 1 to 5. It would be important to disect more precisely as to whether the N-terminal EHFLPRK residues define a functional motif and if they participate in MAD1 binding. Also in the region preceding the KEN-box in animal and yeast species, several residues that are phosphorylated by BUB1 kinase have been identified in CDC20 (e.g., residues S_41_, S_72_, S_92_ in the human CDC20 protein; Tang et al., 2004). Such post-translational modification is important for the inhibition of APC/C^CDC20^ ubiquitin ligase activity (Tang et al., 2004). AtCDC20.1 and all other isoforms have a lesser number of serine residues in the region preceding the KEN-box motif and only one in a similar position (e.g., -22 with respect to the KEN motif, which corresponds to S_9_ in AtCDC20.1). Hence, it appears that compared to animal species, the pattern of CDC20 phosphorylation by BUB1 in *A. thaliana* is expected to be very different. The KEN-box motif and the CRY-box are two degradation signals (degrons) that act as dependent degrons of APC/C in complex with its activator CDC20 homolog 1 (CDH1) (e.g., APC/C^CDH1^) at the end of mitosis (Pfleger and Kirschner, 2000; Sczaniecka et al., 2008; Elowe and Bolanos-Garcia, 2022) and only the former is highly conserved in AtCDC20 isoforms. In addition to these degrons, immediately after the KEN-box motif AtCDC20 harbours a D-box motif, a SLiM that has the (RXXL [G/A]X [I/V/L]X) signature. In AtCDC20.1, the D-box motif is defined by the amino acid residues R_75_ to N_83_. The D-box is considered a classical APC/C degron and it was firstly identified in B-type cyclins as necessary and sufficient for the catalytic activity of the APC/C (Yamano et al., 1998). Interestingly, the KEN-box motif and the D-box motif are both present in APC/C substrates (Davey and Morgan, 2016), and often collaborate to ensure an efficient degradation of APC/C substrates (Tian et al., 2012; Izawa and Pines, 2015; Barford, 2020). This is in contrast with the case of the KEN-box and the CRY-box motifs, which are known to function independently from each other (reviewed by Elowe and Bolanos-Garcia, 2022). It would also be interesting to establish whether the KEN-box and RRY-box (assuming the latter is functional) show an independence of roles in the SAC.

In the human CDC20 orthologue, a serine residue of the CRY-box (C_165_R_166_Y_167_I_168_P_169_S_170_) is phosphorylated by Polo-like kinase-1 (PLK1), a post-translational modification that is required for the timely ubiquitination and destruction of CDC20 (Hyun et al., 2013). At least in mammal oocytes and embryos, the CRY-box has been reported to be an important degron for the destruction of CDC20 in an APC/C^CDH1^ dependent manner (Reis et al., 2006). In *A. thaliana* the CRY-box has degenerated to a R_121_R_122_Y_123_I_124_P_125_Q_126_-box, in addition to the amino acid sequence deviation from a putative CRY motif sequence in *A. thaliana*, the position equivalent of S_170_ in human CDC20 has been substituted by Q_126_ in AtCDC20.1, and this residue is totally conserved in the six AtCDC20 isoforms. However, the +2 position with respect to residue Q_126_ is occupied by a fully conserved Serine (S_128_ in AtCDC20.1). Whether this conserved amino acid residue is a substrate of PLK1 and if this post-translational modification is important for the timely degradation of AtCDC20 isoforms by the ubiquitin-proteasome system (UPS) are important aspects of CDC20 function in plants that remain to be established.

There are numerous examples of SLiMS undergoing important conformational changes upon binding to a protein partner. Often, these involve dramatic disorder-to-order conformational transitions and it can be anticipated a similar mode of binding of AtCDC20 SLiMs to specific protein partners to sustain a SAC response. Future biochemical, biophysical and structural studies on individual AtCDC20 SLiMs will aim to test this hypothesis.

Using an approach that integrated the biochemical analysis of truncated CDC20 constructs with the yeast two hybrid system, an early report described a second MAD2 binding site in CDC20 involving the WD40 domain of the latter molecule (Mondal et al., 2006). The more recent publication of the cryoelectron microscopy (cryoEM) structure of the human APC/C-MCC complex solved at 3.8 Å resolution (PDB ID 6TLJ) adds some support to this view as it shows that a Nitrogen atom of the CDC20 residue E_353_ and Oxygen E1 of the MAD2 residue Q_47_ can establish a stabilising ionic interaction. However, the structure of the human APC/C-MCC complex revealed that the CDC20 region encompassing residues E_126_ to G_135_ are more intimately associated to MAD2, forming a β-strand structure that intercalates with the MAD2 region defined by residues L_153_ to G_170_. The structure of the APC/C-MCC complex also revealed important details of the interactions mediated by CDC20 degrons. Namely, that the region harbouring the IR motif of human CDC20 extends away from the WD40 domain to physically interact with APC/C subunits (Supplementary Figure S3). Given the high conservation of the CDC20 WD40 fold and the IR/LR motif in *A. thaliana* isoforms and CDC20 orthologues of other plant species, it can be anticipated that a similar type of interactions contribute to regulate SAC signalling in the green kingdom.

One intriguing aspect of CDC20 is the early suggestion of a metal binding domain in its WD40 domain and the afirmation that such property affects CDC20 binding to MAD2 (Mondal et al., 2006), which prompted us to carry out an investigation of all CDC20 3D structures alone and in complex that have been reported to date. The crystal structure of the Mitotic Checkpoint Complex from *Schizosaccharomyces pombe* solved at 2.3 Å resolution (PDB ID 4AEZ) revealed the spatial disposition of one tandem of three histidine residues (H_286_-H_288_). The mapping of such residues onto the CDC20 structure showed these histidine residues adopt an orientation that is incompatible with metal ions coordination (Supplementary Figure S4A). An equivalent histidine tandem occurs in human CDC20 (residues H_291_-H_293_) and additional histidine residues are in close spatial proximity to H_291_-H_293_. Namely, the residues H_267_, H_289_, H_300_ and H_301_. Although the mapping onto the 3D structure of human CDC20 of all these histidine residues revealed that four of them adopt an orientation that may favour metal ions coordination (Supplementary Figure S4B), to date no crystal structure of CDC20 from any species that was solved at high resolution; expressed as a histidine tagged fusion; purified by IMAC; and/or crystallised in conditions that included cations at high concentrations such as those deposited in the Protein Data Bank (PDB) under the IDs 4AEZ, 4GGA, 4GGC, 4GGD, and 4N14 has shown that CDC20 binds metal ions at all. In their study, Mondal and collaborators used CDC20 deletion constructs and found that the CDC20 deletion construct lacking the residues Δ218-348 did not bind to Ni-agarose beads while the slightly larger deletion lacking the residues Δ211-355 showed binding to the Ni-agarose beads. Such truncated constructs lack *ca*. 3.2 blades and 3.4 blades of the seven-blades β-propeller fold, respectively, (e.g., nearly half of the WD40 domain was deleted in both cases; data not shown) which is highly likely to result in the expression of CDC20 fragments that are unfolded and prone to stick to agarose/sepharose rather than to coordinate with Ni^+2^ ions. In summary, to date there is no clear evidence of a metal binding requirement for CDC20 for its functions in the SAC and concluded that the assignation to a CDC20 region as a metal binding motif is unjustified and ultimately, misleading.

### AtCDC20.1 WD40 is a globular, monomeric protein

Once a virus titre of high infectivity (1.86 × 10^8^ pfu/ml) was obtained, time course expression tests were conducted in the insect cell lines Sf9 and T. ni in ESF 921 medium at three different multiplicity of infection (MOI) levels and for up to 96 h post infection. As shown in Figures 2A,B, western blot analyses using a human CDC20 antibody which epitope is located within a highly conserved region of the WD40 domain confirmed that AtCDC20.1 WD40 was overexpressed in both Sf9 and T. ni cells. The best expression conditions were achieved in Sf9 cells, MOI 0.1 and 72 h incubation (Figure 2A). Incubation for 96 h following infection at MOI 5.0 resulted in extensive insect cell lysis and degradation of recombinant AtCDC20.1 WD40 in both Sf9 and T. ni cells (Figures 2A,B). The identity of AtCDC20.1 WD40 was further confirmed by western blot using an HRP-conjugated anti-pentahistidine tag antibody (data not shown). Although the levels of expression of AtCDC20.1 WD40 in both insect cell lines were comparable 72 h post-infection at MOI 1.0 and 5.0, they were lower than that seen at MOI 0.1 in Sf9 cells. For this reason, large-scale cultures were carried out in Sf9 cells at MOI 0.1 and cell harvested after 3 days post-infection.

**FIGURE 3:**
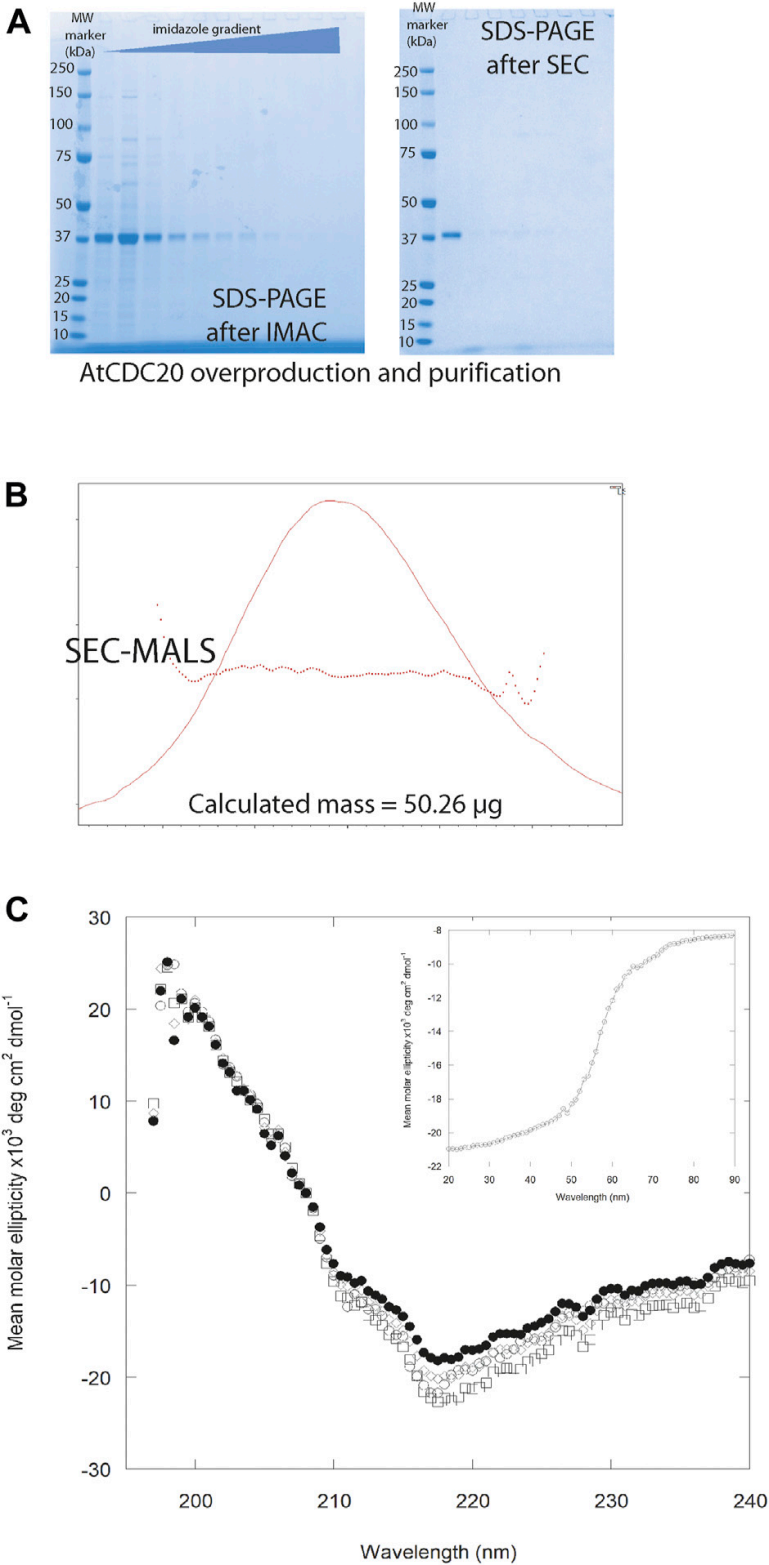
**(A)** AtCDC20.1 WD40 purification by IMAC and after SEC. In both cases, the eluted fractions were compared against the Precision Protein Plus Molecular Weight Marker (M). **(B)** The SEC-MALS profiles confirmed AtCDC20.1 WD40 is a globular protein domain of ca. 40 kDa. **(C)** Far-UV spectra of AtCDC20.1 WD40 at pH 4.0 (○), 6.0 (●), 8.0 (□), and 9.0 (◊). Inset: CD thermal denaturation profile of AtCDC20.1 WD40 recorded at 218 nm (Tm = 56 Celsius). Protein sample prepared in 40 mM sodium phosphate pH 8.0.

The molecular mass of AtCDC20.1 WD40 was very close to that predicted from ProtParam (e.g., 40.09 kDa) (Gasteiger et al., 2005). As shown in Figure 3A, following a step-wise imidazole concentration gradient and IMAC, a protein band of approximately 40 kDa was detected in fractions 3-24, confirming the presence of AtCDC20.1 WD40 in the eluted fractions. Following isocratic chromatographic purification by SEC, AtCDC20.1 WD40 was purified to homogeneity (Figure 3A).

The biophysical characterisation of AtCDC20.1 WD40 by analytical SEC and SEC-MALS analysis showed that AtCDC20.1 WD40 is a globular, monomeric protein in aqueous solutions (Figure 3B). The majority of the WD40 β-propeller domains are characterised by a “velcro” closure that importantly contributes to stabilise the fold (Xu and Min, 2011). Definition of the boundaries of the flexible N-terminal region and the WD40 domain of AtCDC20.1 was informed using a computational biology approach (e.g., homology modelling). Hence it was considered important to establish the stability of the AtCDC20.1 WD40 fragment lacking the N-terminal extension. To this aim, the thermal and pH-dependence stability of AtCDC20.1 WD40 was investigated by CD spectroscopy. As shown in Figure 3C, the CD profile of AtCDC20.1 WD40 in aqueous solutions in the far UV region of the electromagnetic spectrum revealed a folded protein domain with a high content of β-sheet structure that is stable in the pH range 4–9. A lower secondary structure was consistently observed at pH 6.0. Considering the theoretical isoelectric point (pI) of AtCDC20.1 WD40 (pI = 6.53) this behaviour was probably due to protein aggregation. This possibility was then confirmed by direct protein quantification before and after centrifugation of freshly prepared solutions of AtCDC20.1 WD40 at pH 6.0. This manner, an estimated loss of 10–12% of soluble protein due to heavy aggregation was noted. In contrast, AtCDC20.1 WD40 was fairly soluble and stable at pH 4.0 as well as in the pH 8.0-9.0 range. Far UV CD spectroscopy was also used to determine the thermal stability of AtCDC20.1 WD40. The thermal denaturation profile shown in Figure 3C inset indicated a Tm = 56°C, which is comparable to the thermal stability of other seven-bladed β-propeller domains of similar size but diverse function such as the embryonic ectoderm development (EED) protein (Tm = 57°C) (Dong et al., 2019), and lower than a naturally ocurring eight-bladed β-propeller domain, the protein transducin beta-like 1 related (TBLR1) (Tm = 62°C) (Kruusvee et al., 2017), and an artificial eight-bladed β-propeller WD40 domain, the protein IKA8 (Tm = 85°C) (Noguchi et al., 2019).

### Computational analysis of AtCDC20.1 interactions in the SAC

CDC20 plays a pivotal role in the regulation of chromosome segregation and mitotic exit. It exhibits dual, antagonistic functions in the SAC: it functions as an APC/C cofactor and activator (APC/C^CDC20^), but also as a core inhibitor of the APC/C in response to improper kinetochore-microtubule attachments (Alfieri et al., 2016; Yamaguchi et al., 2016; Curtis and Bolanos-Garcia, 2019; Watson et al., 2019). For the latter role, CDC20 associates with BUBR1, MAD2 and BUB3, to form the Mitotic Checkpoint Complex, MCC, (CDC20^MCC^). The MCC binds and inhibits APC/C^CDC20^, forming a large complex called APC/C^CDC20^–MCC that contains two copies of CDC20. High-resolution cryo-EM data of APC/C^CDC20^–MCC complexes revealed important details of APC/C regulation with an unprecedented level of detail (Alfieri et al., 2016; Yamaguchi et al., 2016). The structures showed that the MCC interacts with APC/C^CDC20^ implicating a region close to the APC/C-CDC20 binding interface. The cryo-EM structures also revealed that the MCC component BUBR1 functions as a pseudosubstrate inhibitor that extends around the two CDC20 molecules in such a way that it occupies all degron-binding sites on both molecules, resulting in the allosteric regulation of APC/C ubiquitin E3 ligase activity (Supplementary Figure S3). Although MCC^CDC20^ interaction with the APC/C is mainly mediated through BUBR1 and APC/C^CDC20^, two additional contacts contribute to further stabilise the complex. Namely, the C-terminal IR tail of CDC20 of the MCC binds to a site on APC8A that is structurally equivalent to the APC/C^CDH1^ C-box binding site on APC8B (Chang et al., 2015).

Interestingly, a previous study has shown that five AtCDC20 isoforms can be expressed in yeast cells and establish different types of interactions with other proteins of the SAC-APC/C signalling axis (Kevei et al., 2011). For instance, these authors noted that the AtCDC20.1 and AtCDC20.2 isoforms but not the AtCDC20.3, AtCDC20.4 and AtCDC20.5 isoforms could bind to the APC/C subunit APC10. They also reported a strong interaction between AtCDC20.1 and AtCDC20.2 with MAD2 and BUBR1/MAD3 and a comparatively weaker binding to BUB3.1, while the AtCDC20.5 isoform showed binding to MAD2 but not to BUBR1/MAD3 and BUB3.1. Moreover, AtCDC20.3 and AtCDC20.4 showed no binding to BUBR1/MAD3 and BUB3.1 under the same experimental conditions (Kevei et al., 2011). Such observations argue in favour of distinctive roles for some AtCDC20 isoforms, with some redundancy of function in the regulation of plant cell development such as the case of AtCDC20.1 and AtCDC20.2, while others (AtCDC20.3, AtCDC20.4, AtCDC20.5 and possibly AtCDC20.6) may mediate unique functions due to the differential discrimination of specific interaction partners. The recent identification of a truncated yet functional human CDC20 variant that lacks the flexible N-terminal segment but harbours an intact WD40 domain (Tsang and Cheeseman, 2021) raises the intriguing possibility that *AtCDC20.6 (At5G27945)* is a gene encoding for a similarly N-terminal truncated yet functional CDC20 protein. The question of how exactly sequence variations of AtCDC20 isoforms affect CDC20 functions remains as a fundamental quest in plant physiology that warrants further studies.

**FIGURE 4:**
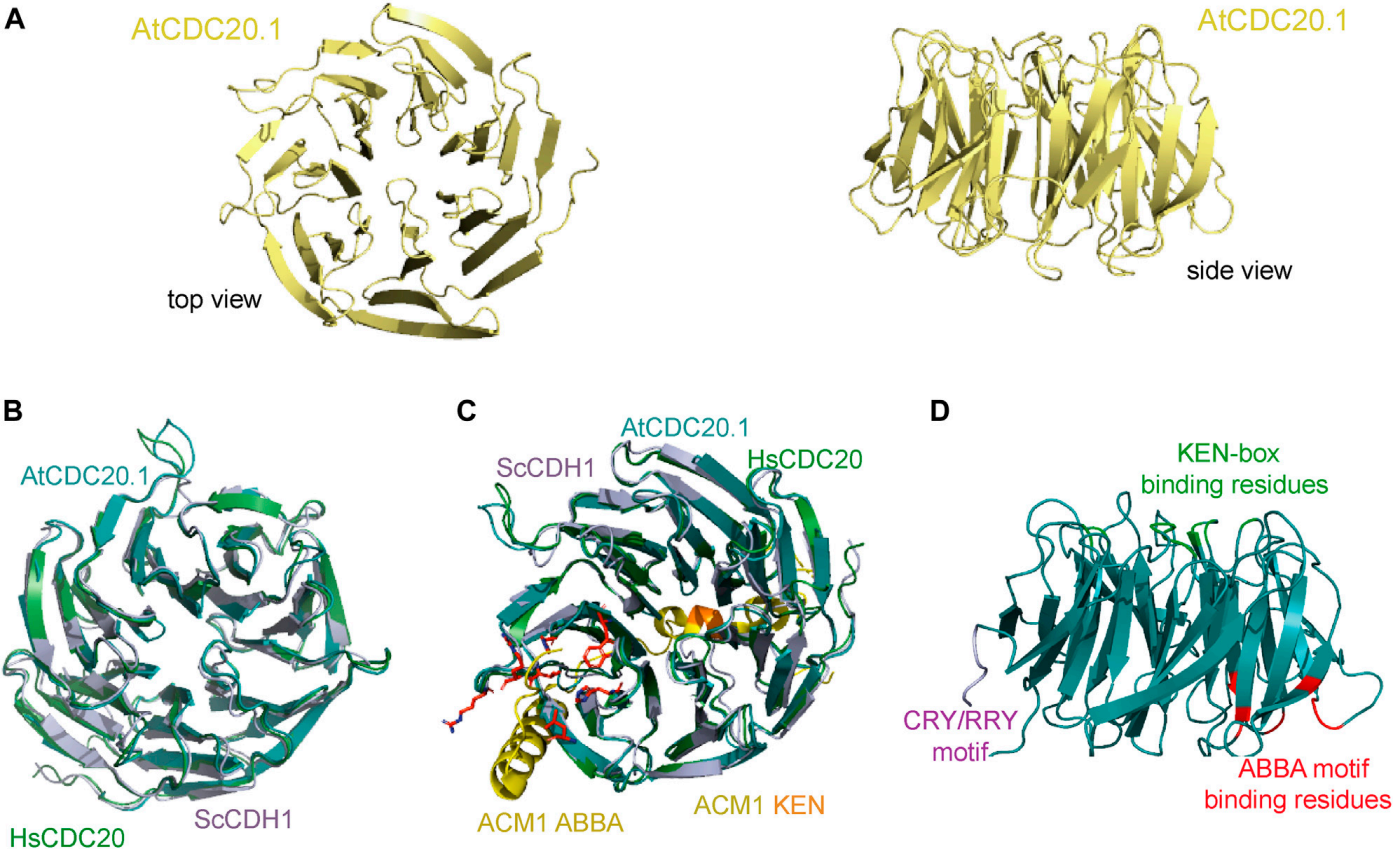
Predicted AtCDC20.1 WD40 3D structure. **(A)** Two views of the AtCDC20.1 3D structure model in a ribbon representation. The model was generated by comparative modelling using Phyre2 (Kelley et al., 2015). **(B)** Structure superposition of AtCDC20.1 WD40 structure model (cyan) with human CDC20 WD40 (green, PDB ID 4GGC), and CDH1 WD40 from budding yeast (purple, PDB ID 4BH6) reveals a high 3D structure conservation of CDC20 from various species and yeast CDH1. **(C)** Structure superposition of CDH1 in complex with an ACM1 fragment containing the KEN-box and ABBA motifs (shown in yellow, PDB ID 4BH6) and CDC20 from human (shown in green, PDB ID 4GGC) and the *A. thaliana* 3D structure model shows the predicted mode of interaction of AtCDC20.1 with interaction partners of this protein that contain these motifs and mediate SAC signalling. The AtCDC20.1 residues predicted to bind the ABBA motif are shown in red in stick and ball representation, while those predicted to bind the KEN-box are highlight in orange. **(D)**. Cartoon illustrating the mapping of the CRY/RRY motif (shown in purple) and a simplified view of the regions containing the residues that are implicated in binding the KEN-box (green) and ABBA motif (red) of CDC20 interaction partners. The binding of AtCDC20.1 to the latter SLiMs is anticipated to be critical for the proper segregation of chromosomes upon cell division in *A. thaliana*.

The overall high amino acid residue conservation in AtCDC20.1 WD40 compared to human and animal orthologues span the entire WD40 domain and enabled the generation of a 3D structure model of this domain using a homology modelling approach (Figure 4A). The predicted high 3D structure similarity of AtCDC20.1 WD40 with the CDC20 WD40 orthologues from human and budding yeast (Figures 4B,C, respectively) suggests a conservation of function in mitosis regulation.

Our 3D structure model of AtCDC20 WD40 suggests that the top face of the β-propeller domain is the region mediating binding to the KEN-box motif of CDC20 interaction partners, implicating the AtCDC20 residues D_139_D_140_F_141_Y_142_Q_267_N_286_N_288_A_316_Q_360_R_404_ and likely to occur in a similar fashion to that observed in the crystal structure of human CDC20 in complex with a peptide that mimics the KEN-box motif of BUBR1 (Protein Data Bank, PDB, ID 4GGD). In human CDC20, the KEN-box binding region involves the residues D_184_Y_185_Y_186_. This triad directly binds to the side chains of the KEN-box motif residues E_27_ and N_28_ of BUBR1, triggering the adoption of an autoinhibited conformation in APC/C^CDC20^. A similar inhibitory mechanism of substrate recognition by highly conserved residues that are located throughout the WD40 domain (Figure 4D) can be expected to operate in *A. thaliana*.

The ABBA motif is another class of SLiM that is present in certain substrates and inhibitors of the APC/C and was initially identified in the CDH1 inhibitor APC/C^CDH1^ modulator 1 (ACM1) from budding yeast (Martinez et al., 2006; Burton, et al., 2011; Di Fiore et al., 2015). This SLiM is defined by a six-residue (Fx [ILV][FY]x [DE]) signature and is common to vertebrate cyclin A, BUBR1 and BUB1, and ACM1p (hence the ABBA acronym) (Di Fiore et al., 2015; Di Fiore et al., 2016; Hein et al., 2021). CDC20 binding to proteins containing the ABBA motif such as BUB1 and BUBR1 is required for a proper SAC response. The amino acid residues of AtCDC20.1 that configure the ABBA motif binding region include H_196_L_217_W_233_H_236_V_252_R_253_ while the CDC20 residues implicated in binding the KEN-box motif are D_139_D_140_F_141_Y_142_Q_267_N_286_N_288_A_316_Q_360_R_404_. Of all the aforementioned SLiMs, the ABBA motif binding residues of AtCDC20 showed the highest sequence variation, with two key hydrophobic residues (Y_240_ and Y_279_ in human CDC20) replaced by histidine residues (H_196_ and H_236_, respectively).

The crystal structure of the APC/C regulator CDH1, a protein that also adopts the β-propeller fold, in complex with an ACM1 fragment containing the KEN and ABBA SLiMs revealed that the ACM1 KEN-box motif binds to a shallow groove at the side of the β-propeller of CDH1, between the β-blades 1 and 7, and that the ACM1 ABBA motif adopts an extended conformation and binds to the inter-blade groove between β-blades 2 and 3 of the CDH1 WD40 domain (Figure 4C). Superposition of the 3D structure model of AtCDC20.1 with yeast CDH1 and human CDC20 supports the notion of similar molecular recognition of KEN and ABBA SLiMs in *A. thaliana*. Interestingly, the AtCDC20.3 and AtCDC20.4 isoforms do not harbour putative degrons, a feature that strongly suggests their timely degradation is not mediated by the APC/C. Future studies should aim to understand the extent of association of AtCDC20 isoforms with the APC/C as to whether the interactions have a differential effect on MCC formation and APC/C activation and inhibition.

### AtCDC20.1 is distributed in different subcellular pools

**FIGURE 5:**
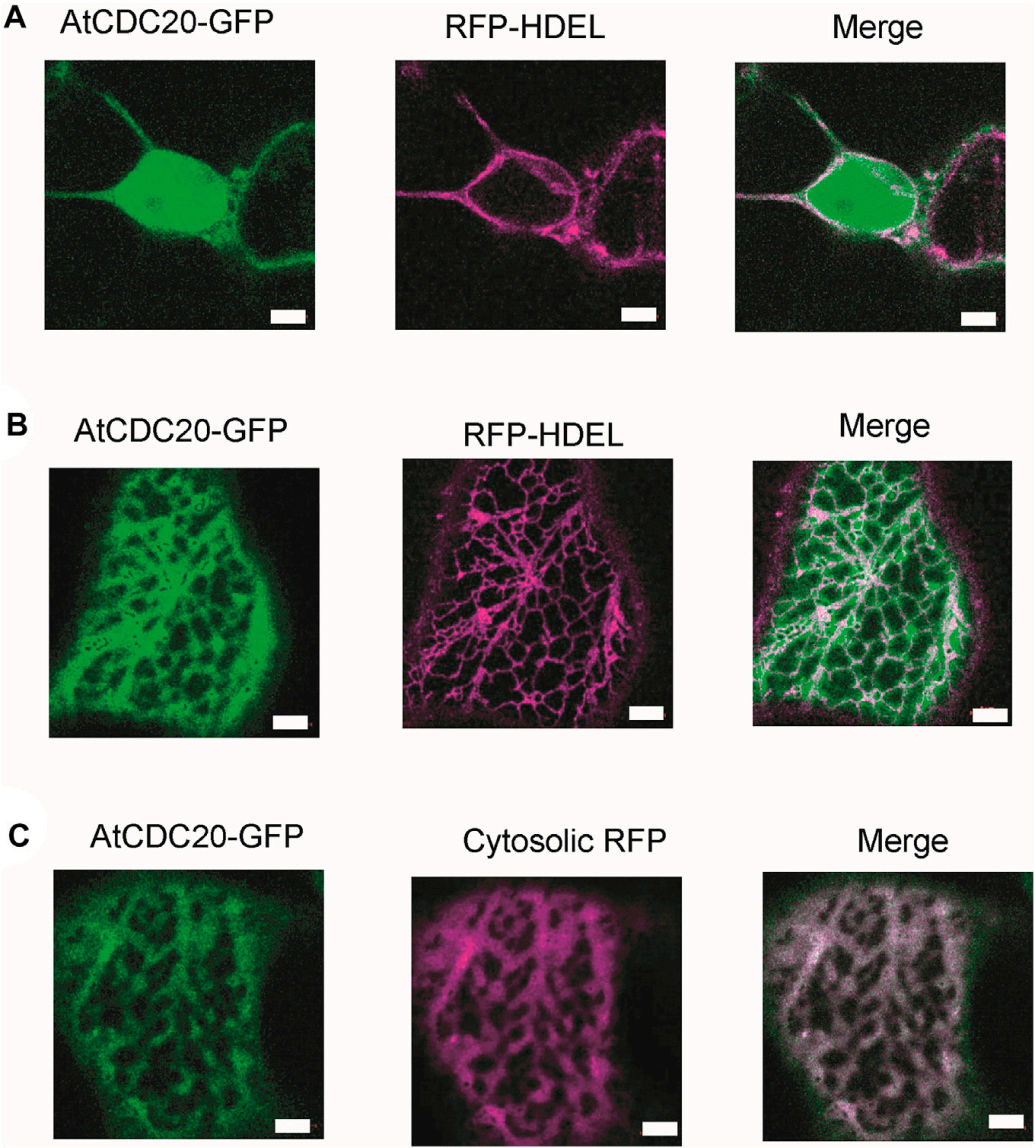
AtCDC20.1 subcellular localisation. AtCDC20-GFP together with suborganellar markers is transiently expressed in tobacco leaf epidermal cells via Agrobacterium-mediated gene expression. AtCDC20.1-GFP is co-expressed with the ER lumenal marker RFP-HDEL and visualised on the nucleus **(A)**, showing labelling in the nucleoplasm and ER **(B)**. AtCDC20.1-GFP co-localises with free RFP labelling the cytosol **(C)**. Example images from *n* = 3 with at least 5 technical replicas each. Size bars = 5 µm.

Previous reports have suggested that certain plant SAC proteins including BUBR1, BUB3, and MAD2, exhibit a peculiar intracellular localisation, accumulating in the kinetochore and spindle microtubules in cells arrested in metaphase (Caillaud et al., 2009). One plausible explanation for this observation is the fact that in plant mitotic cells, the formation of an acentrosomal pro-spindle assembly starts prior to nuclear envelope breakdown (Vos et al., 2008). In such scenario it is possible that SAC proteins are distributed between the kinetochore and spindle microtubules, which is consistent with the notion that plant chromosomes are continuously associated to microtubules (Caillaud et al., 2009). Interestingly, mutants of the *Cdc20.1* gene isoform exhibited malformed meiotic spindles in *Arabidopsis* (Niu et al., 2015), opening the possibility CDC20 also exhibits a subcellular distribution similar to BUBR1, BUB3 and MAD2. Moreover, a previous study using Arabidopsis protoplasts reported a mostly nuclear localisation of some but not all AtCDC20 isoforms (Kevei et al., 2011). One limitation of the study was the lack of *bona fide* cytosol localisation markers. These findings together with the extent of amino acid residue conservation in AtCDC20.1 SLiMs and the anticipated mode of interaction with other protein components of the SAC to regulate chromosome segregation, prompted us to investigate the subcellular localisation of full length AtCDC20.1 in a tobacco epidermal leaf cell expression system. To this aim, AtCDC20.1 fused to green fluorescent protein (GFP) was co-expressed with the ER lumenal marker RFP-HDEL (Figures 5A,B) as well as free RFP labelling the cytoplasm (Figure 5C). Protein localisation was visualised by confocal microscopy. Full length AtCDC20.1 localised to the nucleoplasm (Figure 5A) and did not co-localise with an ER marker (Figure 5B) but with cytoplasmic RFP (Figure 5C) unequivocally indicating that AtCDC20.1-GFP was localised to nucleoplasm and cytoplasm. The observed subcellular localisation of full length AtCDC20.1-GFP (Figure 5) was similar to the recently reported localisation of CDC20 from rice, where transient expression in rice protoplasts of CDC20-GFP fusions were mainly localised to the nuclei and to a lower extent in the cytoplasm (Lin et al., 2022).

## Closing remarks

Full length AtCDC20.1 harbours several conserved SLiMS that play key roles in the control of chromosome segregation and shows a subcellular localisation similar to that observed in other organisms such as animals and fungi. At the same time, AtCDC20.1 and other AtCDC20 isoforms lack SLiMs that are important and highly conserved in animal and yeast species such as a putative MAD1 binding motif and CRY-box degron as well as substrate phosphorylation sites of BUB1 and PLK1 kinases. The C-terminal region of AtCDC20.1 as well as the other five isoforms is mostly organised as a seven-blade β-propeller domain that in most cases shows conserved structural features important for the establishment of productive protein-protein interactions that control the timely and accurate segregation of the genetic material during cell division. AtCDC20.1 WD40 domain was found to be monomeric and stable in aqueous solutions, enabling future biochemical and structural biology studies of AtCDC20.1 WD40-dependent interactions. Given the conservation of MAD2 and APC/C recruitment SLiMS, it can be anticipated an overall conservation of the mode of association of AtCDC20 with other proteins to form the MCC and the interaction of MCC^CDC20^ with the APC/C to regulate SAC signalling in *A. thaliana* and other plants. Whether AtCDC20 isoforms can bind directly to MAD1 using a different SLiM and to what extent it is phosphorylated by BUB1 and PLK1 are important aspects of CDC20 roles in plants that require further investigations. Last but not least, the existence of multiple CDC20 isoforms in *A. thaliana* and the varied extent of conservation of functional motifs may account for some of the differences of SAC signalling between plants and animals.

## Data Availability

The raw data supporting the conclusions of this article will be made available by the authors, without undue reservation.
