# Supplementary figures and images for: Biochemical, biophysical, and functional characterisation of the E3 ubiquitin ligase APC/C regulator CDC20 from Arabidopsis thaliana
Source: Front Physiol. 2022 Jul 25;13:938688. doi: 10.3389/fphys.2022.938688 (PMC9357983; doi:10.3389/fphys.2022.938688)

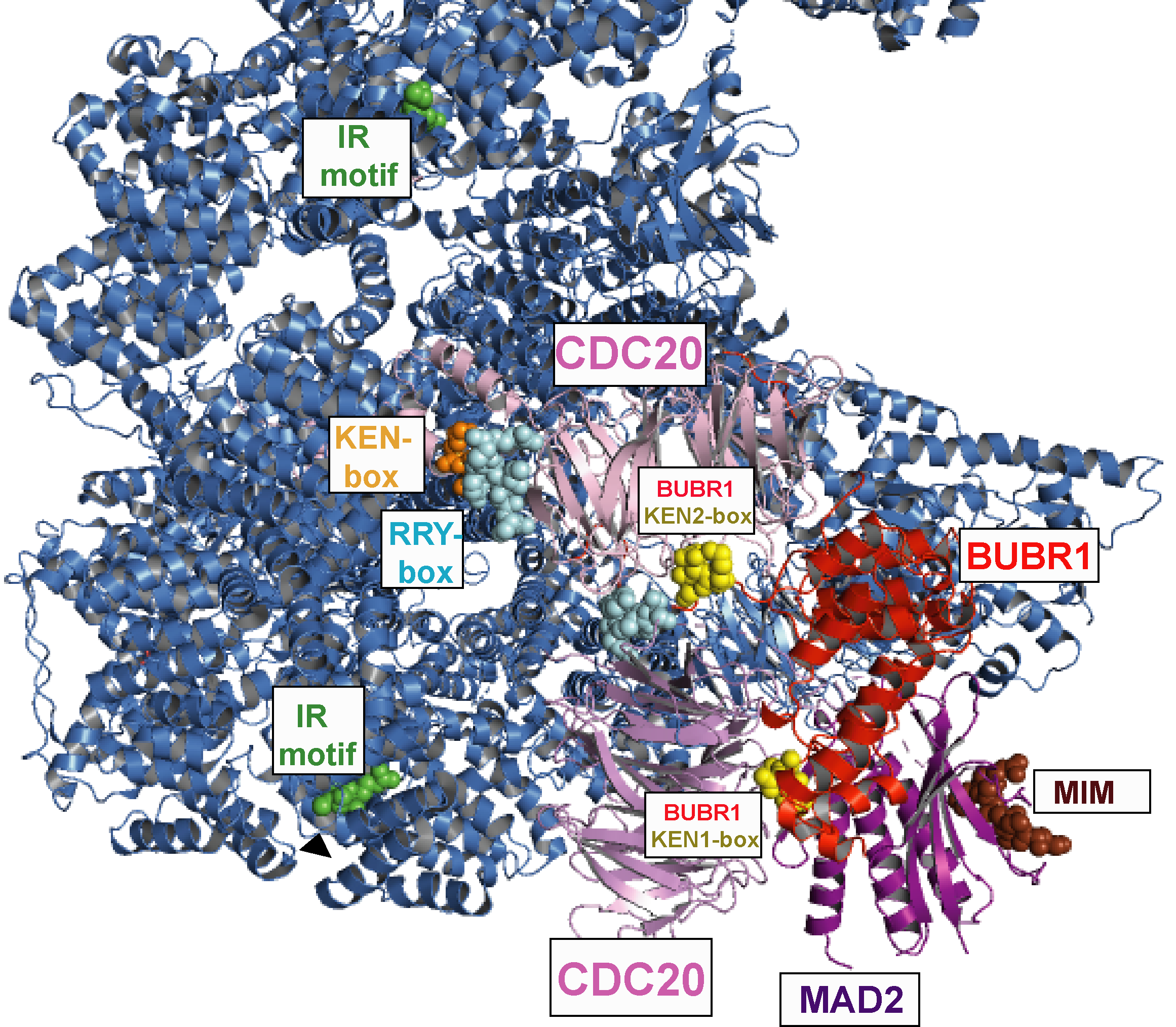

Supplement: Supplementary file 1 [file Image3.TIF]

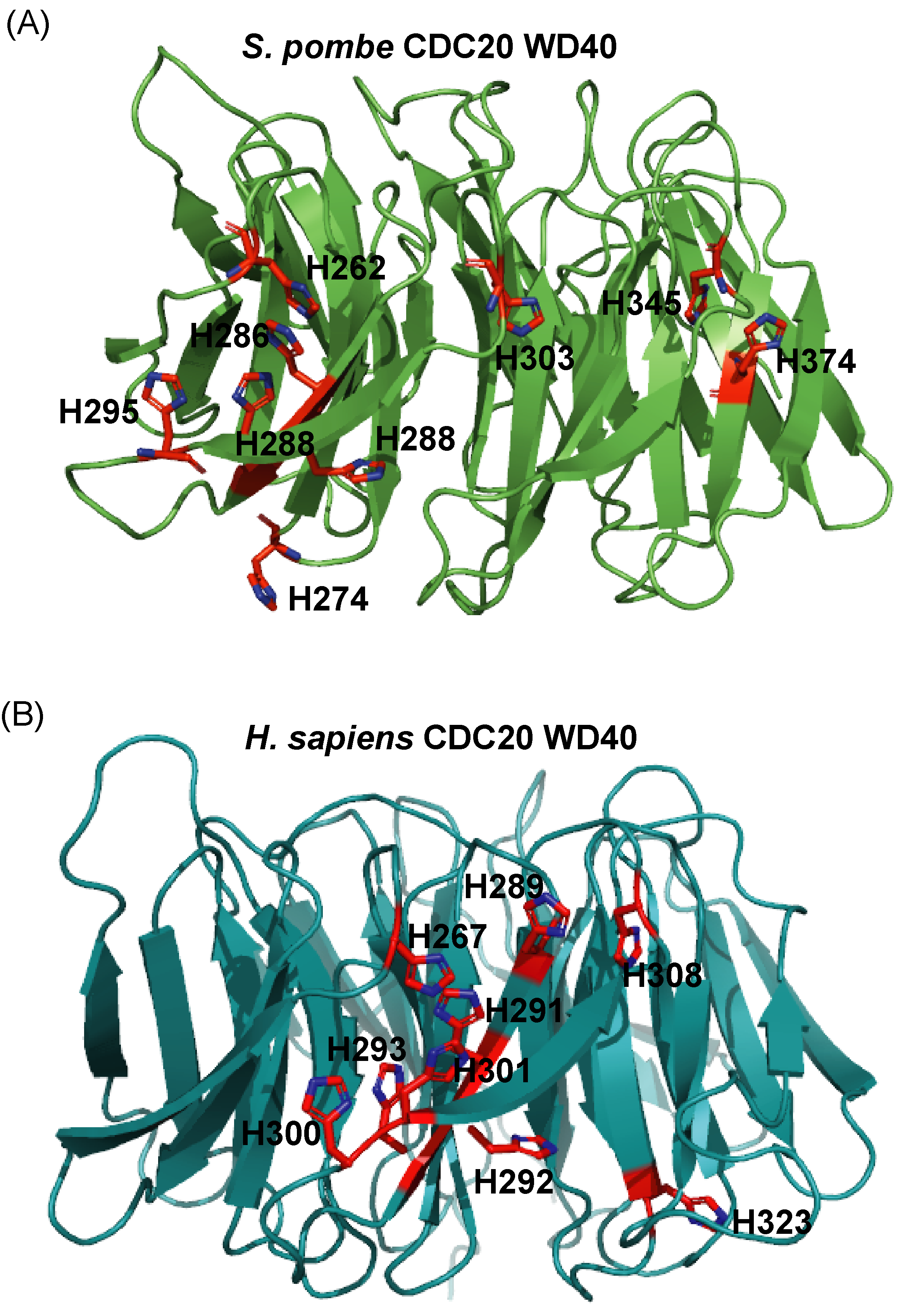

Supplement: Supplementary file 2 [file Image4.TIF]

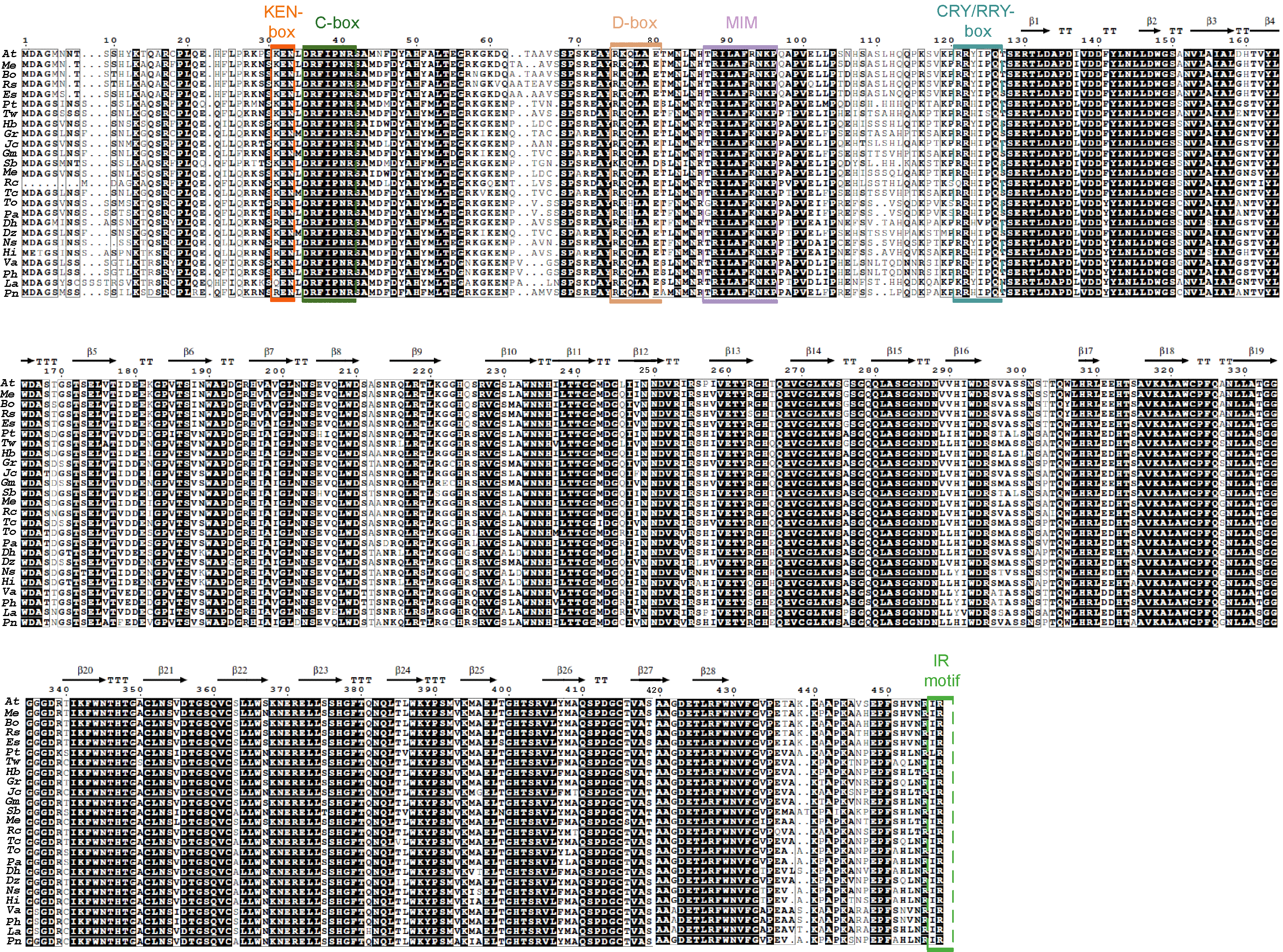

Supplement: Supplementary file 3 [file Image2.TIF]

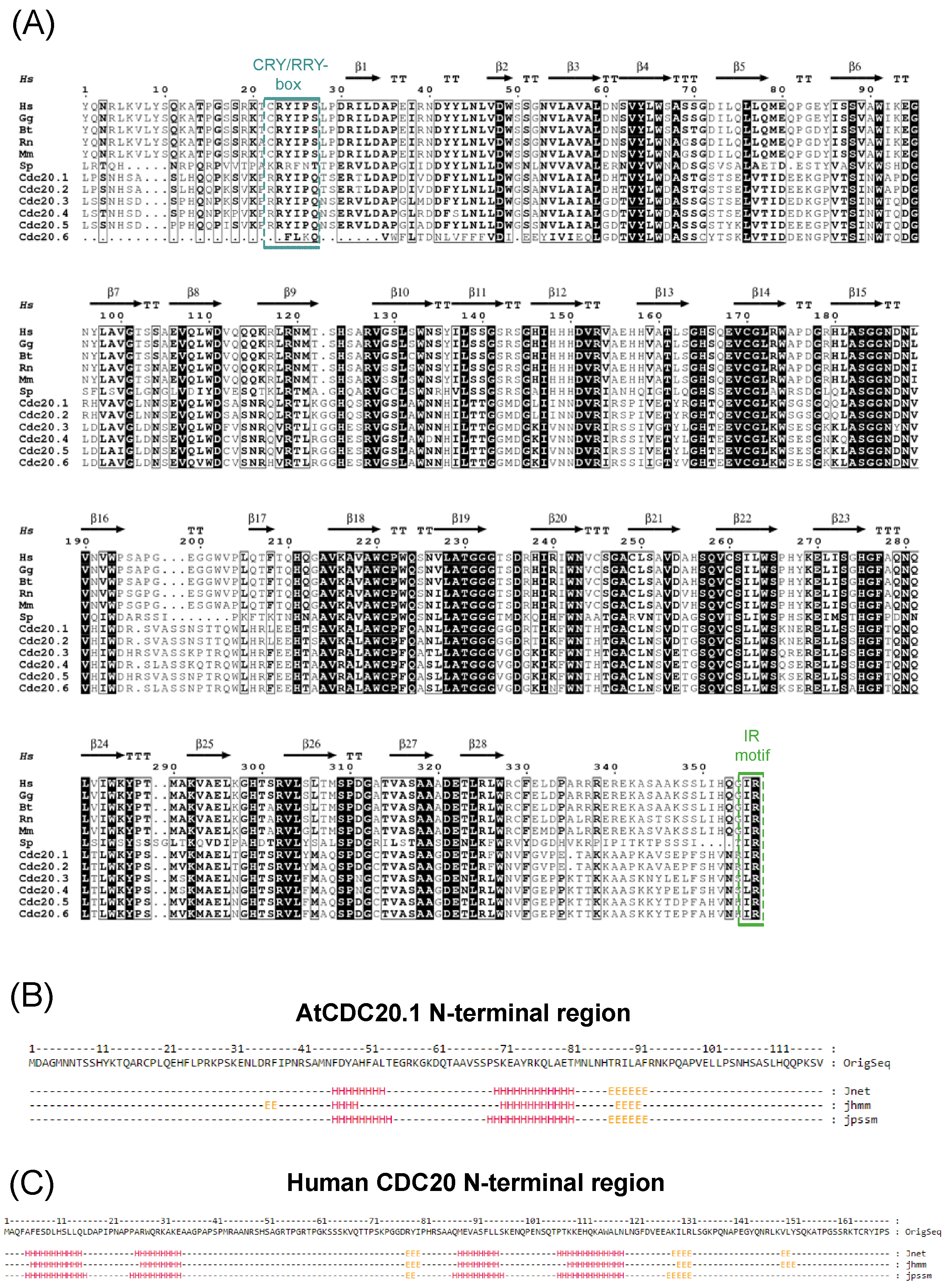

Supplement: Supplementary file 4 [file Image1.TIF]
